# Preliminary Effects of a Mobile Interactive Supervised Therapy Intervention on People Living With HIV: Pilot Randomized Controlled Trial

**DOI:** 10.2196/15702

**Published:** 2020-03-27

**Authors:** Yan Pang, James Steven Molton, Wei Tsang Ooi, Nicholas Iain Paton, Hong-Gu He

**Affiliations:** 1 Alice Lee Centre for Nursing Studies National University of Singapore Singapore Singapore; 2 University Medicine Cluster National University Health System Singapore Singapore; 3 Department of Medicine, Yong Loo Lin School of Medicine National University of Singapore Singapore Singapore; 4 School of Computing National University of Singapore Singapore Singapore

**Keywords:** antiretroviral treatment, highly active, human immunodeficiency virus, medication adherence, mobile application

## Abstract

**Background:**

As people living with HIV infection require lifelong treatment, nonadherence to medication will reduce their chance of maintaining viral suppression and increase the risk of developing drug resistance and HIV transmission.

**Objective:**

This study aimed to evaluate the efficacy of a mobile app, Mobile Interactive Supervised Therapy (MIST), for improving adherence to oral HIV medications among HIV-infected adults in Singapore.

**Methods:**

We conducted a two-group pilot randomized controlled trial (RCT) with a process evaluation, in which 40 HIV-infected participants with once-daily medication regimes were recruited from a public tertiary hospital in Singapore and randomly assigned equally to either the intervention (receiving MIST and routine care) or control (receiving routine care only) groups. The intervention lasted for 2 months. The outcome of antiretroviral therapy (ART) adherence was measured by a 7-day recall self-report (SR), pill count (PC), an electronic medical device—Medication Event Monitoring System (MEMS)—and a mobile app—MIST (for the intervention group only). In total, 20 participants from the intervention group were interviewed at the end of the intervention to assess the acceptability of MIST. Data were collected at baseline and at 1-month and 2-month postintervention.

**Results:**

All participants had excellent medication adherence at baseline (median 100, IQR 100-100). The use of MIST did not result in a significant improvement in ART adherence when measured by the SR, PC, and MEMS, as compared with the control group at 1-month (*P* values >.99, .86, and .74, respectively) and 2-month (*P* values=.80, .84, and .82, respectively) postintervention. ART adherence also did not improve in each group over the same period. MIST was perceived to be a beneficial tool based on the process evaluation results.

**Conclusions:**

Although MIST did not enhance medication adherence to HIV treatments, mainly owing to the ceiling effect, it was perceived to be beneficial among the participants of this study. Our process evaluation provided useful data to further develop MIST for bigger and long-term mobile phone app–assisted intervention RCTs in the future.

**Trial Registration:**

ClinicalTrials.gov NCT03794648; https://clinicaltrials.gov/ct2/show/NCT03794648

## Introduction

### Background

HIV infection is a global public health issue. An estimated 36.9 million people were living with HIV in 2017 [1]. In the same year, 7982 residents of Singapore were living with HIV, with 434 being new cases [2]. In people living with HIV, who require lifelong treatment, nonadherence to medication not only reduces their chance of maintaining viral suppression but also increases the risk of developing drug resistance and HIV transmission [3-6]. To achieve viral suppression, a patient needs to maintain an adherence level of at least 90% throughout the treatment period [6-8]. However, there are challenges to meeting this target. A meta-analysis showed that only 62% of patients of a pooled study aged older than 18 years taking prescribed highly active antiretroviral therapy reported an adherence rate of ≥90% [9]. There are no available antiretroviral therapy (ART) adherence data in Singapore.

With phone technology evolving, mobile phones and smartphones are now able to incorporate many creative features such as recording and transmitting high-quality videos and telemedicine capabilities. There have been a growing number of mobile app reminders supporting patients’ adherence to medication in the market; however, there is a lack of robust evidence to establish their efficacy. To date, the quality of these supporting systems has been variable [10,11]. In addition, current evidence supporting the effectiveness of mobile phone app–based interventions in enhancing adherence among people living with HIV is unclear because of limited evidence and the heterogeneity of the study populations, treatments, and ART adherence measurements [12-17].

### Aims

This study aimed to examine the effects of a mobile phone app, Mobile Interactive Supervised Therapy (MIST), for improving adherence to oral HIV medications among HIV-infected adult patients in Singapore and to gather user experiences to develop the intervention for large scale randomized controlled trials (RCTs) in the future.

### Hypotheses

The hypotheses were as follows:

1. When compared with the control group, patients in the MIST intervention group will have better HIV medication adherence rates at the 1-month and 2-month follow-up time points.
2. When compared with baseline, patients in the MIST intervention group will have better HIV medication adherence rates at the end of the study.

## Methods

### Design

A two-group pretest and posttest RCT design was used. Adult patients infected with HIV (n=40) were recruited from a public hospital in Singapore. Recruited participants were randomly assigned into either of the two groups: the intervention group or the control group.

### Participants

All adults infected with HIV who attended their routine clinic appointments in a public hospital in Singapore from March to June 2018 were approached to participate in this study. The inclusion criteria included those who were (1) aged ≥21 years, (2) taking a once daily regimen of HIV medications, (3) able to take pills orally, (4) willing and able to give informed consent, and (5) able to read and speak English or Chinese. Participants were excluded if they (1) were unable to operate a mobile phone or had an active tuberculosis infection that required directly observed therapy during the study period, (2) had substance use such as methamphetamine use, (3) had visual, speech, or hearing impairment despite the use of aids, (4) had a known medical history of psychiatric disorder(s) and were seeking any form of psychiatric treatment, (5) had a known medical history of cognitive impairment, (6) were fully dependent on a caregiver for taking medications, (7) were pregnant at the time of recruitment and data collection, (8) had a terminal illness such as cancer or late stage cardiovascular disease, or (9) had experienced bereavement within the past 6 months.

### Sample Size Determination

On the basis of pragmatic considerations of the pilot study, a sample of 40 participants, with 20 in each group, was decided a priori [18-20]. All 20 participants in the intervention group were invited for the final process evaluation. There was no dropout during the follow-up period.

### Randomization

**Figure 1:**
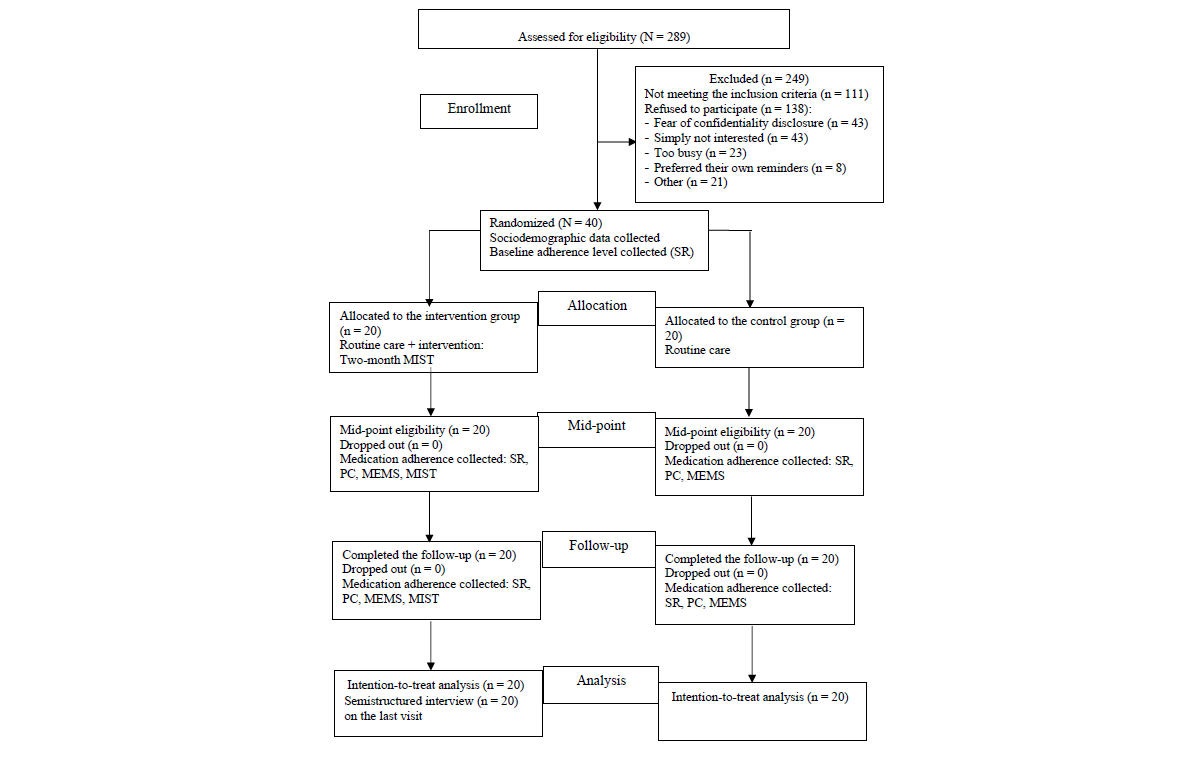
The consolidated standards of reporting trial chart. MEMS: Medical Event Monitoring System; MIST: Mobile Interactive Supervised Therapy; PC: pill count; SR: 7-day recall self-report.

After obtaining written consent, baseline sociodemographic (age, gender, ethnicity, and education level) and clinical data (ART regimen and duration), phone operation technical skills, and a 7-day recall of HIV medication adherence data were collected, and participants were randomly assigned to two groups: the intervention group (receiving the MIST intervention plus routine care) or the control group (receiving routine care only) in a 1:1 allocation ratio. Block randomization with *length 4* was used [21] to ensure a balanced representation in each group [22]. Participants were each asked to open an opaque, sealed envelope with a piece of cardboard inside indicating their randomly allocated groups and were assigned in a successive order according to their enrollment sequence. Through these processes, randomization and allocation concealments were ensured [23-25]. The detailed workflow is described using the Consolidated Standards of Reporting Trial (CONSORT) flowchart (Figure 1).

### Mobile Interactive Supervised Therapy Development and Intervention

**Figure 2:**
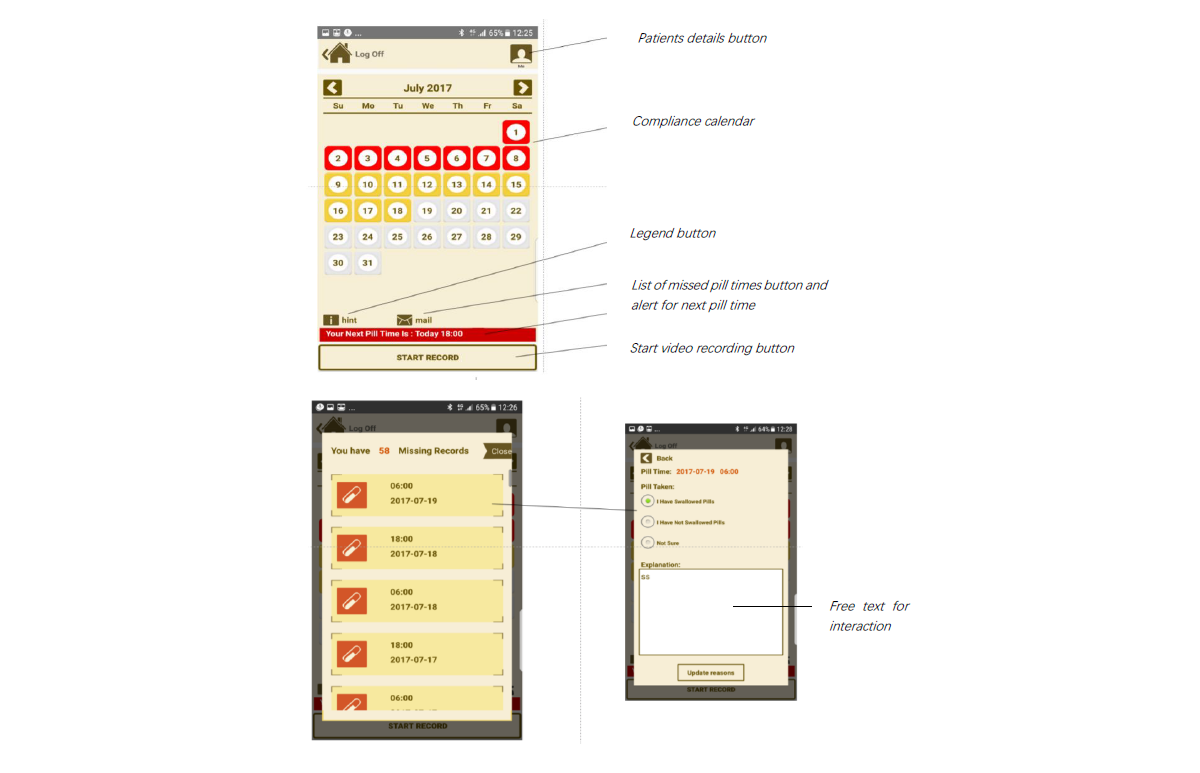
Screenshots of Mobile Interactive Supervised Therapy features.

MIST was developed based on the Theory of Planned Behavior. The MIST system consisted of three components: electronic reminder notifications from the health care system to the patient, transmissions of a daily video of pill-taking from the patient to the health care system, and a color-coded calendar tracker to allow the patient to compare his or her adherence data on any given day with the past week, month, and overall. Screenshots of the MIST features can be found in Figure 2. The previous prototype was developed for both Android and iOS operating systems. Our past pilot study using the earlier prototype showed that MIST was useful in measuring adherence and was acceptable to 42 healthy volunteers [26]. However, as MIST was still a pilot system focusing on producing a functional system, some bugs were identified during pilot testing. Further work was needed to improve the design and usability of the system to ensure the system was robust across a range of users. Since then, MIST has been further developed including fixing bugs such as video upload failure and app instability and was tested among a wide spectrum of phone models during 2016 to 2017. In addition, a reward system was incorporated to incentivize adherence. Before use in this study, the new version was tested in 10 healthy volunteers to ensure technical feasibility.

Participants in the intervention group were asked to use MIST, which comprised integrated Web- and mobile phone–based components. The Web component of the system allowed the administrator to set the time of pill taking each day and to review the submitted videos. The time of the reminder was tagged to the participants’ existing daily ART dosing schedules. Once a daily pill dose time was set, the Web system sent pill reminders (via an SMS or a push notification) to the participant’s mobile phone 15 min before the pill time, right on the pill time, and half an hour after the pill time until a video was received. If no response was received within half an hour after the pill time, the notifications would stop, and the video would be logged as *missing*. When the participants logged their medications on time (between 15 min before and after pill set timing), the frequency of reminders decreased. The Web system stored and uploaded date- and time-stamped videos onto a secure cloud server linked only to a subject number. Successfully uploaded videos were automatically deleted from the phone. Participants who were randomized to the intervention group received one Xiaomi 4A phone (using the Android system) with the MIST app already installed. The standardized phone allowed the app supporter to rapidly resolve any technical issues experienced by participants.

A research assistant (RA) reviewed the uploaded videos daily to ensure that these were of acceptable quality and confirmed pill ingestion. Participants who failed to send a video on time or sent a video that did not permit a verification of pill ingestion were required to provide a reason in their next log-in. If no reason was provided, the RA would contact the participant the next day and ask for an explanation.

The calendar cell colors were synchronized with color codes: a green-colored cell indicated medication adherence, a red-colored cell indicated that the medication was not taken, and a yellow-colored cell indicated that the video was pending the reviewer’s grading. The participants could track their compliance by tapping on each cell of the calendar matrix. There was a built-in reward system that served to reward positive behavior. A reward of Singapore $5 (approximately US $4) was given for every five consecutive successful videos that verified pill intake. The balance could be accumulated and stored in the virtual wallet module of the MIST app and paid to the participants at the final study visit. A maximal reward of Singapore $60 (approximately US $43) would be paid to the MIST users upon completion of the 2-month study.

The participants in the intervention group received a step-by-step guided tour of all the MIST app features via face-to-face demonstrations. All participants verbally indicated that they were confident in using MIST after the demonstrations.

### Outcome Measures and Instruments

The HIV medication adherence rates during the 2-month study period were measured with a combination of objective (pill count [PC], Medication Event Monitoring System [MEMS], and MIST) and subjective (7-day recall self-report [SR]) measures as suggested by the literature [27,28]. We considered taking at least 95% of the prescribed medication as a cut-off to define good adherence in this study as suggested by the literature [29,30].

#### 7-Day Recall Self-Report

Participants were asked about the number of HIV medications they missed in the preceding 7 days at the baseline visit and during the two follow-up visits (1-month visit and 2-month visit). The self-reported medication adherence rate was calculated by subtracting the number of prescribed doses and the number of self-reported missed doses and dividing it by the number of prescribed doses in the past 7 days.

#### Pill Count

The HIV adherence rate was calculated by subtracting the number of prescribed doses in the past 30 days and the number of missed doses in the past 30 days and dividing it by the number of prescribed doses in the past 30 days [31].

#### Medication Event Monitor System

MEMSCap Medication Event Monitoring System, developed by Aardex Group, has a pill bottle with a special cap that contains integrated microcircuits to record the date and time whenever a patient opens a bottle, which wirelessly transfers dosing data when used in conjunction with an MEMSCap reader. A secure, Web-based data platform, AARDEX, processes the transferred adherence data using validated algorithms and presents the information in easy-to-interpret graphs and tables [32-34]. The medication adherence rate is calculated based on the number of times the MEMSCap has been opened divided by the number of times the MEMSCap is scheduled to be opened.

#### Mobile Interactive Supervised Therapy App

Our previous work has demonstrated that MIST is able to accurately measure medication adherence among healthy volunteers in a small pilot study [26]. The ART adherence rate measured by MIST is calculated based on the number of videos received confirming pill intake plus the number of videos not received due to technical problems divided by the number of videos expected during the 2 months.

### Process Evaluation

Acceptability and perceptions on the use of the MIST app were assessed during the final study visit. The RA, who was bilingual in English and Chinese and trained in qualitative research, conducted the semistructured interviews. The interview questions were developed based on the research aims in addition to literature review. Two experts were invited to review the interview guide’s contents upon its development, and minor revisions were made thereafter. The interview was pilot-tested on 3 healthy volunteers to check the appropriateness of the questions as well as the interview process. Further revisions were made to make the questionnaire understandable. The interview mainly addressed five areas: perceptions of the overall MIST app user experience, perceived usefulness of each feature, strengths and weaknesses of MIST, interest in continuing to use MIST in the future, and recommendations for further improvements. A total of 20 individual face-to-face interviews were conducted, lasting for 3 to 13 min.

### Study Procedure

Data collection commenced after ethical approval had been obtained. The primary physicians of the potential recruits were responsible for introducing the participants to the RA. The RA passed a short list of potential recruits to their primary physicians during their scheduled clinic appointments. The primary physicians would briefly introduce the study to their patients. Those who showed interest were then introduced to the RA who would further explain the study to them in a separate private room. Only after obtaining a written consent would the potential participant be recruited into the study. This was followed by a collection of demographic and clinical data and baseline data via a self-administered questionnaire before randomization took place. Outcomes were measured at the following time points for all participants from the two groups: (1) 1 month after the intervention (posttest 1) and (2) 2 months after the intervention (posttest 2).

The RA set up a unique account that was password protected for each participant in the intervention group to log into the MIST app at the baseline visit. The MIST app was activated in the provided Xiaomi 4A mobile phones, and the participants were provided with detailed face-to-face instructions on how to use it. The same RA was responsible for the collection of all data, including process evaluation interviews.

### Data Analysis

Quantitative analyses were performed using the Statistical Package for the Social Sciences (IBM, Version 21.0 [35]).*P*<.05 was considered statistically significant. Descriptive statistics were used to describe demographic data, clinical data, and baseline self-reported medication adherence rates for both groups. Either the chi-square test, Fisher exact test, or the Mann-Whitney *U* test was used to assure comparability between the intervention and control groups.

To answer hypothesis 1, the Mann-Whitney *U* test was used to examine differences in ART adherence rates as measured by the 7-day recall SR, PC, and MEMS between the two groups at the 1-month and 2-month visits.

To answer hypothesis 2, the Wilcoxon signed-rank test was used to compare ART adherence rates (measured by PC, MEMS, and with or without MIST) at different time points (1-month and 2-month visits) for each group, and the Friedman test was used to compare the ART adherence rates (measured by SR) at baseline and the two follow-up visits.

Qualitative data from the interviews were analyzed using content analysis. The audio-taped interview data were transcribed verbatim by the RA to capture nonverbal nuances. A range of highlighter colors were used to highlight segments of data portraying similar ideas and meaning. Subsequently, data extracts that have been coded with the same color were collated and organized into categories. The refined potentially repeated patterns across the entire data were, then, transferred to a separated word document. The initial codes were reviewed again by the RA and another investigator, and related codes were collated to form subthemes [36]. Different opinions were discussed until a mutual agreement was reached between the two investigators [37]. Rigor was ensured through credibility, transferability, dependability, and confirmability in this study process [38].

### Ethical Considerations

Ethics approval for the study was received from the institutional review board before commencing the study (Ref: 2017/00150). All participants provided written consent for audio-recordings of the interviews. Confidentiality of the data and voluntary participation were explained to all participants.

## Results

### Participants’ Demographics and Clinical Data and Group Comparison

Among the 40 participants who participated in the study, 38 (95%) were male and 25 (63%) were Chinese, 8 (20%) were Malay, 3 (8%) were Indian, and 4 (10%) were from other ethnic groups. The median age was 37.45 (IQR 30.07-44.95) years. In total, 21 of 40 (53%) participants were doing professional work. Nearly half of them (19/40, 48%) completed university or higher education. Most were single (32/40, 80%), lived with family (29/40, 73%), lived in a Housing and Development Board flat or a studio apartment (36/40, 90%), and had home Wi-Fi (29/40, 73%). The median ART durations at the baseline were 25.15 (IQR 12.23-48.03) months and 22.35 (IQR 8.93-43.28) months for the control and intervention groups, respectively. Taking two types of tablets of HIV medication was the most commonly reported regime (16/40, 40%) among the participants, followed by one type of tablet (11/40, 28%), three types of tablets (8/40, 20%), and at least four types of tablets (5/40, 13%). The vast majority of the participants were very confident in operating smartphones (34/40, 85%). As shown in Multimedia Appendix 1, baseline characteristics were similar between the control and interventional groups except for types of ART medication (*P*=.01).

### Comparison of Outcomes

As shown in Table 1, there were no significant differences in the medication adherence rates between the intervention and control groups at the follow-up time points. In addition, neither of the measures reported significant differences in ART adherence rates between the two groups across the 2-month period (Table 2).

**Table 1:** Comparison of median antiretroviral therapy adherence rates between the control group and intervention group at the 1-month and 2-month follow-up visits (N=40).

Follow-up visit and ART^a^ adherence rate (%) measurement		Control group, median (IQR)	Intervention group, median (IQR)	*P* value
**1 month**				
	Self-report	100 (100-100)	100 (100-100)	>.99
	Pill count	100 (100-100)	100 (100-100)	.86
	MEMS^b^ median	100 (93-100)	100 (93-100)	.74
**2 months**				
	Self-report	100 (100-100)	100 (100-100)	.80
	Pill count	100 (100-100)	100 (100-100)	.84
	MEMS median	97 (93-100)	97.5 (92-100)	.82

^a^ART: antiretroviral therapy.

^b^MEMS: Medication Event Monitoring System.

**Table 2:** Comparison of median antiretroviral therapy adherence rates at different time points for each group (N=40).

ART^a^ adherence rate (%) measurement		Baseline, median (IQR)	1-month visit, median (IQR)	2-month visit, median (IQR)	*P* value
**Self-report**					.22^b^
	Control group	100 (100-100)	100 (100-100)	100 (100-100)	
	MIST^c^ intervention group	100 (100-100)	100 (100-100)	100 (100-100)	
**Pill count**					.07^d^
	Control group	N/A^e^	100 (100-100)	100 (100-100)	
	MIST intervention group	N/A	100 (100-100)	100 (100-100)	
**MEMS^f^**					.63^d^
	Control group	N/A	100 (93-100)	97 (93-100)	
	MIST intervention group	N/A	100 (93-100)	97.5 (92-100)	
**MIST**					
	MIST intervention group	N/A	96 (87-100)	94 (87-99)	.25^d^

^a^ART: antiretroviral therapy.

^b^Friedman test.

^c^MIST: Mobile Interactive Supervised Therapy.

^d^Wilcoxon signed-rank test.

^e^N/A: Not applicable.

^f^MEMS: Medication Event Monitoring System.

### Process Evaluation

All 20 participants from the intervention group completed the semistructured interviews. Among 20 participants who were interviewed, the majority of them were extremely or very confident (18/20, 90%) in operating a mobile phone. Participants were aged between 27 and 52 years. Most were males (n=19) and Chinese (n=13). Half of them were doing professional work, 2 were doing skilled work, while 7 were unemployed, and 1 was a student. Slightly over half of them completed university or higher education, 35% (7/20) completed a college, and the remaining 10% (2/20) received a secondary school education. The majority of them were single (16/20, 80%) and lived with family or friends. Findings related to the MIST app user experiences were grouped into six categories: (1) perceived ease of use, (2) benefits of MIST, (3) MIST app features preferences, (4) MIST app dislikes, (5) future willingness to use MIST, and (6) suggestions for MIST improvement.

#### Category 1: Perceived Ease of Use

When asked about their overall experience with the MIST app, half of the participants reported that the app was user-friendly.

#### Category 2: Benefits of Mobile Interactive Supervised Therapy

Most of the participants considered MIST to be beneficial. It not only reminded and motivated them to take their ART medications punctually and consistently every day but also helped in situations when they were tired and forgot to take their medications. They also agreed the color-coded calendar helped them to track their medication-taking histories. In all, 2 participants verbalized that receiving cash helped to lighten their financial burdens.

#### Category 3: Mobile Interactive Supervised Therapy App Feature Preferences

When asked for their preferred app features, the participants voted almost in equal numbers for each feature, that is, SMS reminders, color-coded calendars, and the reward system were voted by 7, 6, and 5 participants, respectively.

#### Categories 4 and 5: Mobile Interactive Supervised Therapy App Dislikes and Willingness for Future Utilization

Some participants revealed that there were certain aspects of the MIST app that they disliked, and these included the need for an internet connection, app glitches and instability, limitation to mobile phones using the Android platform, extra burdens (such as recording themselves taking their medications), and privacy concerns. Due to these concerns, among the 20 participants who were interviewed, only 6 (30%) participants expressed their willingness to use MIST in the future.

As there was stigma associated HIV infections, the participants tried to minimize receiving SMS reminders or taking their medications in public to prevent attracting attention. For instance, 1 participant switched off the provided phone as he was unsure what the contents of the incoming SMS reminders would look like. Another participant was concerned that recording himself taking the medication would trigger curious onlookers. A third participant suggested that MIST should have an option that allowed the users to change the app icon image as the default icon might inadvertently reveal their HIV status.

#### Category 6: Suggestion for Mobile Interactive Supervised Therapy Improvement

Most of the participants suggested making the app features more user-friendly. The MIST app should be made available for all types of phones. They also suggested alternative reward methods and methods to track the medications taken. Some thought that the app should incorporate a fun element to engage future users.

## Discussion

### Principal Findings

The pilot trial on the use of MIST, which followed patients infected with HIV for 2 months, did not show any significant improvements in ART adherence rates. However, this finding needs to be interpreted with caution as this was just a pilot study and the sample recruited were those who had good medication adherence. On the basis of the process evaluation, MIST was perceived to be beneficial among our participants. Our process evaluation provided useful data to further develop MIST for bigger and long-term mobile phone app–assisted intervention RCTs in the future. The reason for the lack of improvement was the ceiling effect. Before the study, both groups of participants were already adhering to their medications. For example, the median 7-day self-recalled ART adherence at the baseline was 100% in both groups, leaving no room for them to improve their adherence further. Given a longer enrollment time, we would have liked to target subjects who were at high risk of nonadherence. However, our subject recruitment was limited by the small number of newly diagnosed patients annually. In addition, the recruitment was hampered by perceived stigma in people living with HIV and concern that the intervention might compromise confidentiality and privacy. After a slow start of having only 3 patients recruited, we expanded our eligibility criteria to include those who had no adherence problems as well. However, the rejection rate was still nearly 48%. The study ended with most of the participants already having good ART adherence. The selection bias was likely to contribute to our observation that patients with poor ART adherence might be also those who were less interested to participate in the trial. In support of our assertion of the ceiling effect, one study [15] did not have the ceiling effect as it only enrolled participants who reported less than 95% adherence and demonstrated adherence improvements following their mobile phone app use. The second possible reason for the lack of improvement in our study is that most of our participants were already on ART for about 1 year, as evidenced by the median ART duration (IQR) for the control group of 25 months (IQR 12-48 months) and the intervention group of 22 months (IQR 8-43 months) before the study enrollment. The need for lifelong daily ART adherence would have developed among them a well-established daily medication-taking routine [39]. In all, two previous phone technology–assisted adherence RCTs [40,41], which targeted only patients newly initiated onto ART, demonstrated the effectiveness of SMS or SMS combined with counseling in improving medication adherence. The results highlighted the importance of initiating the intervention at the early stage before the participants establish medication-taking habits [40,41].

Despite the absence of an effect of MIST on ART adherence among the participants who had 100% adherence rates at baseline, our qualitative findings demonstrated that the MIST app was beneficial to most participants. They perceived MIST to be user-friendly. It is interesting to note that although most of our participants did not forget to take their medications, the reminders were especially helpful for others who did shift work or who were busy with other things at the time their medications were due. This finding is consistent with a previous RCT [39], in which some participants who did not benefit from reminders would nevertheless recommend it to someone who already had a fixed HIV medication–taking routine [39]. Some perceived the color-coded calendar tracking system to be more useful compared with a conventional tracking tool, such as a diary or pillbox, or even no tracking tool. The visual calendar medication-taking tracking feature helped our participants to save time and, more importantly, motivated them to continue their good medication-taking behaviors. Our study findings are consistent with previous research, which suggested that an app with health-related behaviors and goal tracking features, such as medication-taking behaviors, was perceived as valuable [42].

We believe the following reasons contributed to the reluctance of most of the participants (14/20) in continuing to use MIST in the future. First, the availability of the latest prototype version was limited to the Android operating system as it was still being optimized across all Android operating system phones during the study period. Owing to this limitation, we had to provide each participant with a Xiaomi 4A model phone that contained the MIST app. Failure to carry the phone, turn it on, or have the battery charged prevented the reminders from serving their desired purpose. Second, occasional app glitches and instability were barriers that discouraged participants from continuing to use the MIST app. Some participants claimed that they would rather have taken their medications than wait for the app to start working. Third, some participants found video recording while taking their medications burdensome, which might not be sustainable as HIV requires lifelong medication adherence. However, in spite of this, all 20 participants maintained their commitments and continued using MIST until the end of the study. On the contrary, a trial [13] observed a 25% reduction in the use of their Heart2HAART mobile phone app during a 3-month trial period. In their study, participants in the Heart2HAART group were expected to respond to randomly generated daily medication prompts, including questions about HIV medication side effects and substance cravings during the study period. They concluded that user fatigue might have contributed to app usage reduction [13]. Our study finding stood in contrast to the previous study findings [13], possibly because of the cash reward system that was built into MIST, whereas there was no incentive system in the previous study [13]. As having cash incentives might not be a practical measure in the long term, future studies should consider alternative incentive methods.

Although the MIST app is password protected, some of the participants expressed concerns about possible disclosures of their HIV status during the interviews. Compounding the privacy concern was the bulky electronic monitor device—MEMS—which might have triggered onlookers’ curiosity and a loss of privacy thereafter. Our findings are consistent with findings from other studies. For example, a global qualitative review study revealed that the acceptability of mobile technology–assisted interventions that aimed to improve ART adherence among people living with HIV could be affected by privacy and confidentiality issues [43]. In view of the findings of this study, we will optimize MIST to make sure that it is more secure. We will modify the MIST prototype based on other suggestions of our participants. For instance, the app icon should be made customizable. MIST should be optimized to market-level quality, meaning that it should be made more sensitive and less glitchy and it should be downloadable for use in other operating systems. We will also develop a backup plan in case the MIST app fails to work. For example, verbal reports or SMS as an alternative to report adherence.

### Limitations of the Study

This study had several limitations. First, owing to pragmatic considerations, the small sample size of 40 participants might have failed to detect a significant difference in medication adherence (ie, type 2 error). Second, we did not collect biological data such as CD4 count and viral load, which are good indicators for treatment success. Third, although this study was conducted in a tertiary hospital, due to the intervention itself, some older people were excluded from this study as they were not confident in using the technology. Blinding was not possible for both participants and researchers due to the nature of the app. Hence, this study might have introduced some response bias. MIST participants might have responded differently owing to social expectation pressure. Fourth, the interview duration was short. However, this was a process evaluation as opposed to an in-depth qualitative study, and all interview questions were straightforward. Furthermore, the majority of potential participants declined to be interviewed owing to stigma. Stigma could also have limited the information that enrolled participants were willing to share during interviews. Finally, because of time constraints, the majority of our participants had good baseline ART adherence resulting in the ceiling effect. The intervention was limited to a 2-month follow-up, and this short follow-up period does not reflect the real-world situation, as HIV requires lifelong commitment and adherence.

### Conclusions

In conclusion, this pilot study showed that MIST was perceived as beneficial by our participants. Future RCTs with a bigger sample size and the use of an optimized and more acceptable version of MIST should be conducted to evaluate the effectiveness of MIST in enhancing medication adherence among people living with HIV, especially those who have poor adherence and those who have just started on ART.

## Appendix

Multimedia Appendix 1 Comparison of the participants’ sociodemographic, clinical, and technical skills data (N=40).

Multimedia Appendix 2 CONSORT-EHEALTH checklist V 1.6.1.

## Abbreviations

ART: antiretroviral therapy
MEMS: Medication Event Monitoring System
MIST: Mobile Interactive Supervised Therapy
NHIC: National Health Innovation Centre
PC: pill count
RA: research assistant
RCT: randomized controlled trial
SR: self-report
