# Supplementary material for: Preliminary Effects of a Mobile Interactive Supervised Therapy Intervention on People Living With HIV: Pilot Randomized Controlled Trial
Source: JMIR Mhealth Uhealth. 2020 Mar 27;8(3):e15702. doi: 10.2196/15702 (PMC7148554; doi:10.2196/15702)
Supplement: Multimedia Appendix 1 [file mhealth_v8i3e15702_app1.docx]

Comparison of the participants’ sociodemographic, clinical, and technical skills data (N=40)

| Characteristics | Total (N=40) | Control group (n=20) | Intervention group (n=20) | *X^2^/Z -value* | *P* value |
| --- | --- | --- | --- | --- | --- |
| **Demographics** | | | | | |

|  | Age (years), median (IQR) | 37.45 (30-45) | 35.50 (28-44) | 37.90 (31-45) | −0.26^a^ | .80^b^ |
| --- | --- | --- | --- | --- | --- | --- |
|  | **Gender, n (%)** | — | — | — | 0.00^c^ | 1.00^d^ |

|  |  | Male | 8 (95) | 19 (95) | | 19 (95) | — | — |
| --- | --- | --- | --- | --- | --- | --- | --- | --- |
|  |  | Female | 2 (5) | 1 (5) | | 1 (5) | — | — |
|  | **Ethnicity, n (%)** | | — | — | | — | 1.87^c^ | .60^d^ |
|  |  | Chinese | 25 (63) | 12 (60) | | 13 (33) | — | — |
|  |  | Malay | 8 (20) | 5 (25) | | 3 (15) | — | — |
|  |  | Indian | 3 (8) | 2 (10) | | 1 (5) | — | — |
|  |  | Others | 4 (10) | 1 (5) | | 3 (15) | — | — |
|  | **Occupation, n (%)** | | — | — | | — | 4.65^c^ | .46^d^ |
|  |  | Unemployed | 10 (25) | 3 (15) | | 7 (35) | — | — |
|  |  | Student | 2 (5) | 1 (5) | | 1 (5) | — | — |
|  |  | Skilled/Technical | 4 (10) | 2 (10) | | 2 (10) | — | — |
|  |  | Clerical work | 1 (2.5) | 1 (5) | | 0 (0) | — | — |
|  |  | Retail/Sales | 2 (5) | 2 (10) | | 0 (0) | — | — |
|  |  | Professional | 21 (53) | 11 (55) | | 10 (50) | — | — |
|  | **Education level, n (%)** | | — | — | | — | 1.87^c^ | .60^d^ |
|  |  | No formal education | 0 (0) | 0 (0) | | 0 (0) | — | — |
|  |  | Secondary school | 4 (10) | 2 (10) | | 2 (10) | — | — |
|  |  | ITE/Polytechnic/Junior college | 17 (43) | 10 (50) | | 7 (25) | — | — |
|  |  | University | 14 (35) | 5 (25) | | 9 (45) | — | — |
|  |  | Postgraduate or higher | 5 (13) | 3 (15) | | 2 (10) | — | — |
|  | **Marital status, n (%)** | | — | — | | — | 4.80^c^ | .91^d^ |
|  |  | Single | 32 (80) | 16 (80) | | 16 (80) | — | — |
|  |  | Married | 5 (13) | 4 (20) | | 1 (5) | — | — |
|  |  | Divorced | 3 (8) | 0 (0) | | 3 (15) | — | — |
|  |  | Widow | 0 (0) | 0 (0) | | 0 (0) | — | — |
|  | **House type, n (%)** | | — | — | | — | 2.11^c^ | .55^d^ |
|  |  | Housing development board flat/studio apartment | 36 (90) | 17 (85) | | 19 (85) | — | — |
|  |  | Private flats/condominium | 2 (5) | 1 (5) | | 2 (10) | — | — |
|  |  | Landed property | 1 (3) | 1 (5) | | 1 (5) | — | — |
|  |  | Others | 1 (3) | 1 (5) | | 1 (3) | — | — |
|  | **Living with, n (%)** | | — | — | | — | 3.23^c^ | .36^d^ |
|  |  | Family | 29 (73) | 14 (70) | | 15 (75) | — | — |
|  |  | Friends | 4 (10) | 1 (5) | | 3 (15) | — | — |
|  |  | Alone | 5 (13) | 3 (15) | | 2 (5) | — | — |
|  |  | Others | 2 (5) | 2 (10) | | 0 (0) | — | — |
| **Clinical data** | | | | | | | | |
|  | **Types of ART^e^ medicine, n (%)** | | —^f^ | — | — | | 11.41^c^ | .01^d^ |
|  |  | One type of tablet | 11 (28) | 1 (5) | 10 (50) | | — | — |
|  |  | Two types of tablets | 16 (40) | 9 (45) | 7 (35) | | — | — |
|  |  | Three types of tablets | 8 (20) | 6 (30) | 2 (10) | | — | — |
|  |  | At least four types of tablets | 5 (13) | 4 (20) | 1 (5) | | — | — |
|  | ART duration (months), median (IQR) | | 24 (12-43) | 25 (12-48) | 22 (9-43) | | −0.42^a^ | .68^b^ |
| **Technical data** | | | | | | | | |
|  | **Network source, n (%)** | | — | — | — | | 1.60^c^ | .45^d^ |
|  |  | Home Wi-Fi | 29 (73) | 13 (65) | 16 (80) | | — | — |
|  |  | Work Wi-Fi | 0 (0) | 0 (0) | 0 (0) | | — | — |
|  |  | Mobile data | 7 (18) | 5 (25) | 2 (10) | | — | — |
|  |  | Home Wi-Fi and mobile data | 2 (10) | 2 (10) | 2 (10) | | — | — |
|  | **Technical skill confidence level, n (%)** | | — | — | — | | 4.14^c^ | .25^d^ |
|  |  | Not at all | 0 (0) | 0 (0) | 0 (0) | | — | — |
|  |  | A little | 1 (3) | 0 (0) | 1 (5) | | — | — |
|  |  | Moderately | 5 (13) | 4 (20) | 1 (5) | | — | — |
|  |  | Very confident | 14 (35) | 5 (25) | 9 (45) | | — | — |
|  |  | Extremely | 20 (50) | 11 (55) | 9 (45) | | — | — |
| **ART adherence** | | | | | | | | |
|  | Self-reported ART adherence rate, median (IQR) | | 100 (100-100) | 100 (100-100) | 100 (100-100) | | 0.00^a^ | 1.00^b^ |

^a^X^2^-value.

^b^Mann-Whitney U test.

^c^Z-value.

^d^Chi-square test.

^e^ART: antiretroviral therapy.

^f^-: not applicable
